# Single-cell Migration Chip for Chemotaxis-based Microfluidic Selection of Heterogeneous Cell Populations

**DOI:** 10.1038/srep09980

**Published:** 2015-05-18

**Authors:** Yu-Chih Chen, Steven G. Allen, Patrick N. Ingram, Ronald Buckanovich, Sofia D. Merajver, Euisik Yoon

**Affiliations:** 1 Department of Electrical Engineering and Computer Science, University of Michigan, 1301 Beal Avenue, Ann Arbor, MI 48109-2122, USA; 2 University of Michigan Comprehensive Cancer Center, 1500 E. Medical Center Drive, Ann Arbor, MI 48109, USA; 3 Program in Cellular and Molecular Biology, University of Michigan Medical School, Ann Arbor, MI; 4 Medical Scientist Training Program, University of Michigan Medical School, Ann Arbor, MI; 5 Department of Biomedical Engineering, University of Michigan, 2200 Bonisteel Blvd, Ann Arbor, MI 48109, USA; 6 Department of Internal Medicine and Comprehensive Cancer Center, University of Michigan, 1500 E. Medical Center Drive, Ann Arbor, MI 48109, USA

## Abstract

Tumor cell migration toward and intravasation into capillaries is an early and key event in cancer metastasis, yet not all cancer cells are imbued with the same capability to do so. This heterogeneity within a tumor is a fundamental property of cancer. Tools to help us understand what molecular characteristics allow a certain subpopulation of cells to spread from the primary tumor are thus critical for overcoming metastasis. Conventional *in vitro* migration platforms treat populations in aggregate, which leads to a masking of intrinsic differences among cells. Some migration assays reported recently have single-cell resolution, but these platforms do not provide for selective retrieval of the distinct migrating and non-migrating cell populations for further analysis. Thus, to study the intrinsic differences in cells responsible for chemotactic heterogeneity, we developed a single-cell migration platform so that individual cells’ migration behavior can be studied and the heterogeneous population sorted based upon chemotactic phenotype. Furthermore, after migration, the highly chemotactic and non-chemotactic cells were retrieved and proved viable for later molecular analysis of their differences. Moreover, we modified the migration channel to resemble lymphatic capillaries to better understand how certain cancer cells are able to move through geometrically confining spaces.

Cell migration is an essential process in angiogenesis, cancer metastasis, wound healing, inflammation, and embryogenesis. In particular, significant attention has been paid to the migration of cancer cells since cancer metastases account for more than 90% of cancer-related mortality^1^,^2^. Cancer metastases result from a multi-step process with significant attrition of viable cells at each step in the metastatic cascade. One such rate-limiting step is the chemotactic migration and intravasation of tumor cells from the tumor stroma to a capillary bed or lymphatic vessels^1^,^2^,^3^,^4^. The study of the intravasation step has been hampered though by the lack of accessible *in vitro* techniques. Additionally, the regulation of certain metastasis-related genes also modulates the occurrence and burden of metastases. Although several genes have been discovered and may be potential targets for therapeutics^5^,^6^,^7^, the study of these metastasis-related genes still largely depends on xenograft or tail-vein injection mouse models, which focus on global differences in large cell populations and require considerable time and expense thereby precluding their adaptation or input into personalized therapy^2^,^4^,^8^. Furthermore, single-cell resolution of mechanical differences and direct visualization are also at present impractical in xenograft-based experiments in which typically only metastatic growth endpoints are assessed rather than the interceding steps. Hence, there is a need to develop *in vitro* devices which can realistically emulate critical steps of the metastatic cascade – especially the confining geometry of intravasation into and migration through blood and lymphatic capillaries – and allow for the direct visualization of the process as well as allowing for the separation and further characterization of cells with differing chemotactic properties^2^,^3^.

Popular long-standing approaches for studying cell motility and invasion *in vitro* such as wound healing and transwell assays have significant limitations^9^,^10^. Wound healing assays present challenges both in the reproducibility of the scratch and in the inability to discern and separate the more motile from the less motile cells within a population^11^. Transwell assays provide quantitative binary motility results in large cell populations, but imaging of the actual migration process of the individual cells is not possible. These fundamental limitations preclude the use of these assays to understand in detail the migration of cancer cells under conditions that more closely mimic steps of the metastatic cascade. Realizing these limitations and taking advantage of modern microfabrication technologies, a number of studies have employed microfluidic channels to study cell migration more effectively^12^,^13^,^14^,^15^,^16^. In some studies, different channel cross-sectional sizes and geometries have been used to study the effects of geometry on cell migration^15^,^17^,^18^,^19^,^20^, while in others the migration channel was filled with hydrogel or extra-cellular matrix components in order to simulate the cancer invasion process through stroma^21^,^22^. In yet other approaches, two or more cell types were co-cultured in microfluidic channels to approximate the cellular diversity in the tissue micro-environment^23^,^24^,^25^. However, these previous microfluidic approaches that study collective migration behaviors lack the concurrent capability to trace in detail a single cell’s behavior, capture migrating cells, and investigate cell population heterogeneity with regards to chemotaxis. Furthermore, the geometry-based studies were not on the biological scale of pre-lymphatics and lymphatic capillaries^15^,^26^,^27^,^28^.

Cellular heterogeneity is a key characteristic of cancer and cancer cell populations are diverse within a tumor mass^1^,^29^,^30^. Due to genetic differences as well as differing epigenetic and metabolic regulation, subgroups of cancer cells in a tumor have distinct growth advantages as the conditions change and thus diverse phenotypes with differing migration and metastatic capability evolve in a tumor mass over time^30^,^31^. As researchers have begun to recognize the importance of cellular heterogeneity contributing to metastasis, several studies have reported on techniques to study the migration behavior of individual cancer cells^32^,^33^,^34^,^35^,^36^,^37^. These prior platforms have a low capture efficiency, typically loading many cells yet only investigating a small portion. This inefficiency proves critically unfavorable for applications that use rare samples such as primary tumor analysis. More importantly, these platforms, as with other microfluidic migration devices, do not allow for the retrieval of cells after the migration assay. This re-harvesting of the cells after the migration assay for further downstream analysis is of the utmost importance for an understanding of the fundamental causes of increased chemotaxis in some cells within a population that is otherwise the same. Although recently one study has demonstrated the separation of a cancer cell line population based on chemotactic phenotype, it did not enrich for increased chemotaxis in the selected subpopulation as compared to the parent population and required the loading of thousands of cells^16^.

Therefore, to overcome these and other limitations, we designed a single-cell migration platform that allows for the post-migration collection and analysis of differing chemotactic subpopulations of cells and that can be modified to geometrically mimic the tight spaces in the pre-lymphatic and lymphatic capillaries that a cancer cell must navigate on its way to metastasize^3^,^26^,^28^. Our platform incorporates a single-cell capture scheme which positions one cell at the entrance of each migration channel, so the chemotactic behavior of each individual cell can be specifically traced and delineated over time. Using this cell positioning technique, assays are performed by monitoring 20 captured cells in each device, making the platform favorable for future use with primary tumor samples and other rare cells in contrast to prior devices. Importantly, after the migration assays, the highly chemotactic cells can be retrieved for further downstream molecular and phenotypic analysis in comparison to the non-chemotactic subpopulation.

We show that the highly chemotactic subpopulation of MDA-MB-231 breast cancer cells selected through the migration assay maintain this migratory phenotype after harvesting and reintroduction to the migration assay. Furthermore, the chemotactic cells have a more mesenchymal morphology compared to the non-chemotactic residual cells and also express significantly greater amounts of the mitogen activator of protein kinase (MAPK) isoform p38γ and Ras-homology (Rho) GTPase isoform RhoC, both critical modulators of mesenchymal motility in MDA-MB-231 cells^38^. Lastly, using our lymphatic capillary *in vitro* mimic, we corroborate our prior *in vivo* results that showed p38γ was necessary for contralateral lymph node metastasis^38^. We customized the migration channels in our device to include choke points on the geometric scale of the constricting lymphatic capillary dimensions *in vivo* in order to allow our study *ex vivo* of the capillary intravasation step of cancer metastasis to the lymph nodes^26^. Using p38γ shRNA knockdown and scrambled vector control breast cancer cells in our newly developed lymphatic-mimetic device, we show that the knockdown p38γ cells are unable to effectively traverse choke points at the critical size of 6 μm × 10 μm, which is the size of lymphatic vessels *in vivo*^26^. Our present device can thus be used to directly visualize one of the critical steps of the metastatic cascade in order to reveal further insights into what molecular underpinnings allow certain cancer cells within a heterogeneous tumor to intravasate into capillaries and subsequently metastasize.

## Results

### Design of the single-cell capture scheme

**Figure 1:**
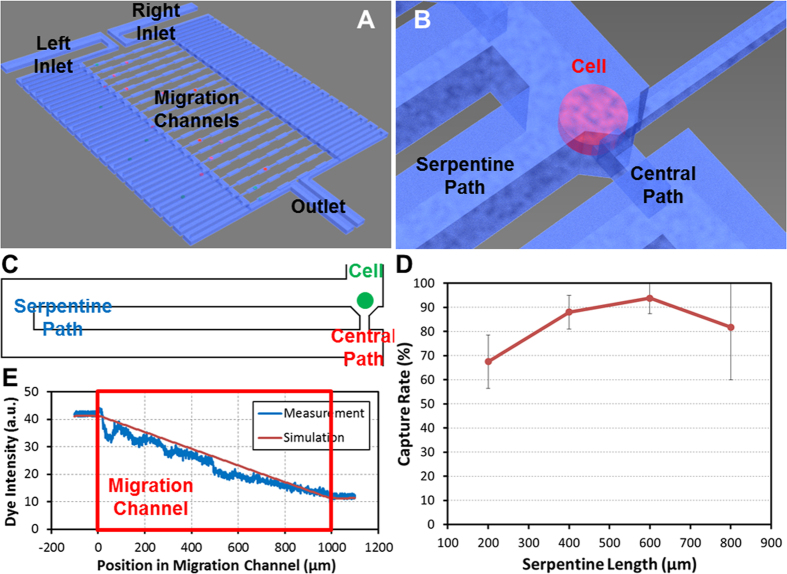
Proposed microfluidic chip for single-cell migration. (**A**) 3D schematic drawing of the chip. The cells are loaded in the left side, and the chemoattractant induces the migration through the migration channels toward the right. (**B**) Enlarged 3D schematic drawing of one cell capture site. (**C**) Schematic of the cell capture principle. (**D**) SKOV3 cell capture rate with different serpentine lengths (N = 4 devices). The optimal length determined from these experiments was 600 μm. (**E**) Concentration gradient of chemicals in the migration channel. The red line indicates the simulation result by COMSOL 3.5. The blue line is the measurement of the fluorescent dye intensity in the migration channel.

The microfluidic device consists of single-cell capture sites and migration channels. Figure 1 (A) shows a schematic diagram of the described chip and fabrication processes. To achieve single-cell resolution, cells are loaded by gravity flow and hydrodynamically captured at each capture site (Fig. 1 (B)). We incorporated the capture area directly adjacent to the entrance of the narrow migration channel, an innovative feature as compared to other devices^39^,^40^,^41^. As shown in Fig. 1 (C), two paths are created: a shorter central path and a longer serpentine path. The flow rate of each path is inversely proportional to its hydrodynamic resistance. A long serpentine structure increases the hydrodynamic resistance (R_S_), so the serpentine flow resistance is larger than that of the central path. Therefore, the serpentine flow (Q_S_) is less than the central flow (Q_C_), and cells will more likely follow the central path. As the opening of the central path is slightly smaller (height: 20 μm, width: 10 μm) than the size of cancer cells (e.g., SKOV3 cells has an average diameter of 14.1 μm, SD ± 3.2 μm) and funnel-shaped, the captured cell consistently plugs the gap and blocks the flow through the central path (cell valving). Thus, the remainder of the cells travel through the serpentine path and are subsequently captured in the downstream capture sites (Fig. S1).

In order to optimize the length of the serpentine path and achieve a high cell capture rate, we simulated the pressure and velocity field for various channel geometries. Fig. S2 shows the simulation of the pressure and flow velocity under varying serpentine lengths ranging from 200 μm to 800 μm. Ideally, the larger the hydrodynamic resistance of the serpentine path is (R_S_, which is proportional to the serpentine length) the higher the capture rate. However, when the serpentine length is longer than 800 μm, the hydrodynamic resistance of the serpentine path (R_S_) becomes so large that the flow velocity drops significantly. In this case, cells may get stuck along the serpentine path resulting in clogging. As a result, there is a large standard deviation of capture rates observed in chips with very long serpentine lengths (Fig. 1 (D)). A similar problem arises under gravity flow when many more than 20 migration channels are incorporated (data not shown). To further optimize the cell capture rate in this asymmetric capturing design, the media volume in the right inlet during loading (80 μL) is slightly less than that in the left inlet (100 μL). The resulting weak gravity flow rightward into the migration channel guides the cells closer to the capture site to increase the capture probability. From simulations, the optimal serpentine length was determined to be 600 μm, to achieve high capture rates of over 94% (capturing nearly exactly one cell per each migration channel) (Fig. 1 (D)). A video demonstrating the cell capturing process is shown in Mov. S1.

The stiffness of the cells of interest is also critical for optimal cell capture. More elastic cells yield higher capture rates since they deform more easily and plug the central path, sealing the capture site better than stiffer cells do. Based on the particular cancer cell types used in the experiments, the geometry of serpentine lengths and path openings were modified to improve capture efficiency, as described in more detail in the supplementary methods. Extensive studies were performed on various cell types including SKOV3, A2780DK, C2C12, MDA-MB-231, and PC3 cell lines, and we have achieved capture rates greater than 85% in all the tested cell types (Table 1). These experimental data demonstrate that the proposed single-cell capture mechanism is reliable and robust for a broad spectrum of cell types, and thus amenable to the study of individual cancer cells’ migration.

### Chemical gradient generation

The migration of cancer cells can be driven by chemotaxis whereby differences in the concentration of growth factors or other chemokines can induce tumor cells to intravasate into the circulatory system^42^,^43^,^44^. To model this *in vivo* condition, the migration channels in our device (width: 40 μm, height: 10 μm, length: 1 mm) are designed to specifically study the movement of cells with a concentration gradient profile generated by diffusion^45^. To generate this chemical concentration profile, serum-free culture media with chemoattractant is pipetted into the right inlet and serum-free culture media is pipetted into the left inlet. Due to the nature of diffusion, the concentration of the chemoattractant in the migration channels increases linearly along the channel from left to right, as simulated in Fig. S3. The generated chemical profile projected in the simulation was confirmed experimentally using a fluorescent dye (Fluorescein 5(6)-isothiocyanate, F3651, Sigma-Aldrich). The fluorescent intensity was measured and plotted in Fig. 1 (E). The measured fluorescent concentration profile agrees with simulation results (COMSOL 3.5), verifying that concentration profiles can be successfully generated. Additional simulations were performed to investigate whether a migrating cell in the channel would affect the gradient profile. A pseudo-cell (10 μm width by 10 μm height and 40 μm length) was added to the model to simulate a potentially blocked channel. However, since the channel cross-section (40 μm by 10 μm) is much larger than that of a cell (10 μm by 10 μm), the gradient was only minimally changed (<2% difference), as shown in Fig. S4.

### Single-cell migration assay

Cancer metastases are caused by a multi-step process which begins with the escape of tumor cells from the primary tumor through the basement membrane and the subsequent intravasation of cancer cells into capillary vessels under the influence of chemoattractants and cellular signals generated by cell-cell junctions^2^,^42^. In order to validate the utility of the fabricated migration chip as an *in vitro* model of migration, we investigated the chemotaxis of SKOV3 ovarian cancer cells toward a higher concentration of hepatocyte growth factor (HGF), which is a well-known chemoattractant across many cell types^46^.

**Figure 2:**
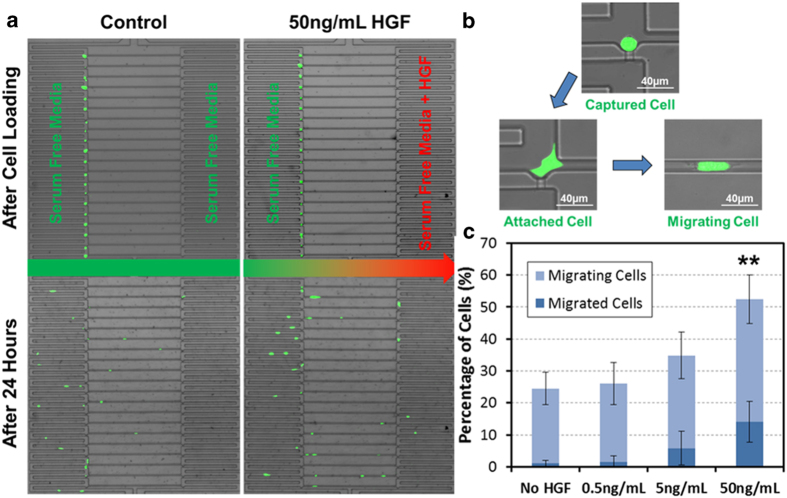
Single-cell migration assay using HGF as a chemoattractant for SKOV3 cells. (**A**) The images of the single-cell migration assay. The upper two images illustrate the single-cell distribution after cell loading (cells were loaded from the left channel). All captured cells are aligned along the left side of the migration channels. The lower two images illustrate the cell distribution after 24 hours without a chemoattractant (lower left, control) and with 50 ng/mL of HGF in serum-free media added to the right inlet (lower right, stimulated). Compared to the control, the HGF induced cells to migrate further to the right. (**B**) The process of cell migration. First, a cell is captured by the hydrodynamic force from the cell solution. After 4–6 hours the cell attaches to the substrate and then it begins to move into the migration channel. (**C**) Result of the chemoattractant assays. The graph illustrates the relative ratio of migrated cells (all the way to the opposite side) and migrating cells (within the channel) vs. HGF concentration. The result confirms that the HGF is a strong chemoattractant for the SKOV3 cells. Data points represent means ± standard deviations (N = 4 devices), ** refers to P < 0.01 compared to the no HGF control.

Figure 2 illustrates the single-cell migration tests in the platform. After cell loading utilizing gravity-driven flow, all the captured SKOV3 cells were positioned at the capture sites along the left side, as shown in Fig. 2 (A). The captured cells attached to the substrate within three hours and chemotaxis was monitored over 24 hours at single-cell resolution, as shown in Fig. 2 (B). After cell loading, media in the right inlet was replaced with serum-free media supplemented with 50 ng/mL HGF, which induces SKOV3 cell migration^47^. Serum-free media without HGF was pipetted into the left inlet, creating a linear concentration gradient of HGF along the migration channel. After 24 hours, we observed that more cells migrated to the right side when exposed to the HGF concentration gradient, while under the control conditions (applying serum-free culture media to both inlets) the cells did not show any directional migration (Fig. 2 (A)). This increase in chemotaxis was dependent on HGF concentration and is plotted in Fig. 2 (C). These data demonstrate that our platform is suitable for studies relying on single-cell chemotaxis as a read-out.

### Selective subpopulation harvesting for downstream cellular heterogeneity analysis

Cellular heterogeneity is a key characteristic of cancer. Subpopulations or even single cells in a primary tumor or within a cancer cell line may have their own distinct phenotype due to genomic mutations or differential genetic and epigenetic regulation^1^,^29^,^30^,^31^. In our single-cell migration platform, we can not only monitor the movement of each cell but also selectively harvest the highly chemotactic and the non-chemotactic subpopulations for downstream analysis after the migration assay. This additional capability grants the opportunity to analyze the intrinsic differences within cell populations which contribute to the observed heterogeneity in motility.

**Figure 3:**
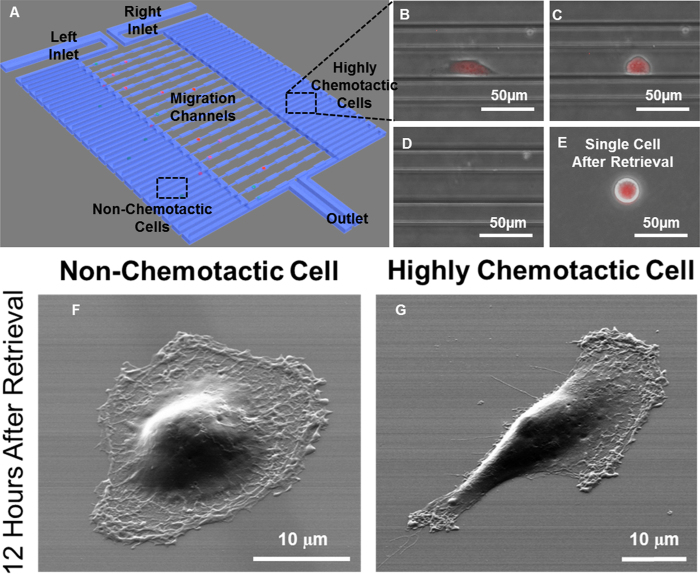
Selective retrieval and downstream analysis of the highly chemotactic cells. (**A**) The schematics for cell retrieval. (**B**) A representative highly chemotactic cell, which has migrated completely through the migration channel within 24 hours. (scale bar: 50 μm) (**C**) After 3 minutes of trypsinization, the cell became rounded in morphology. (scale bar: 50 μm) (**D**) After 5 minutes of trypsinization, the cell was successfully detached and flowed to the right inlet. (scale bar: 50 μm) (**E**) All detached cells were transferred to a 60 mm petri-dish or 96-well plate. (scale bar: 50 μm) (**F**) Scanning electron microscope image of a non-chemotactic cell exhibiting epithelial morphology 12 hours after retrieval. (scale bar: 10 μm) (**G**) Scanning electron microscope image of a highly chemotactic cell exhibiting mesenchymal morphology 12 hours after retrieval. (scale bar: 10 μm).

Although MDA-MB-231 is an aggressive breast cancer cell line, some cells within this line exhibit yet a greater chemotactic potential than others in that we observe not all cells migrate equally toward a chemoattractant stimulant (data not shown). To understand the differences even within a traditionally presumed “homogenous” cell line that could lead to this phenotypic dissimilarity, we sought to collect and further characterize the highly chemotactic MDA-MB-231 population in comparison to the cancer cells that remained on the cell-loading side of the device and were not stimulated by the gradient to directionally migrate. After a migration assay using 10% fetal bovine serum as the chemoattractant, both highly chemotactic and non-chemotactic MDA-MB-231 cells were retrieved as illustrated in Fig. 3 (A–E). Figure 3 (B) shows a highly chemotactic MDA-MB-231 cell, which had migrated all the way to the serpentine channel on the right side of the device within 24 hours. To selectively harvest these cell populations, we must use a different protocol than for cell seeding. For cell loading at the start of a migration experiment, we utilize only gravity-driven flow by adding 100 μL of cell-containing media into the inlets with no liquid in the outlets to achieve a pressure difference of around 50–100 Pa. For cell retrieval, trypsin is flowed from the outlets to the inlets, detaching and directing chemotactic cells to the right inlet and the non-chemotactic cells to left inlet. Additionally, we apply a negative pressure of about 1,000 Pa via pipet bulb on the inlet reservoirs as trypsin flows from the outlet reservoirs. This pressure gradient generates a flow rate strong enough to overcome the slight diameter difference between a cell (~13 μm) and the central path capture site (20 μm × 10 μm) so that the cells deform and flow through the capture channel and toward the collecting inlets, without incurring damage. After 5 minutes of trypsinization under negative pressure, the target cell populations were detached, retrieved from the inlet, and then re-plated into 60 mm petri dishes for recovery and propagation (Fig. 3 (C, D, E)). After 12 hours of recovery, scanning electron microscope images of the retrieved cells revealed that the highly chemotactic cells were more elongated with a distinct mesenchymal morphology whereas the non-chemotactic cells were rounded with an epithelial-like morphology (Fig. 3 (F,G))^48^,^49^,^50^.

**Figure 4:**
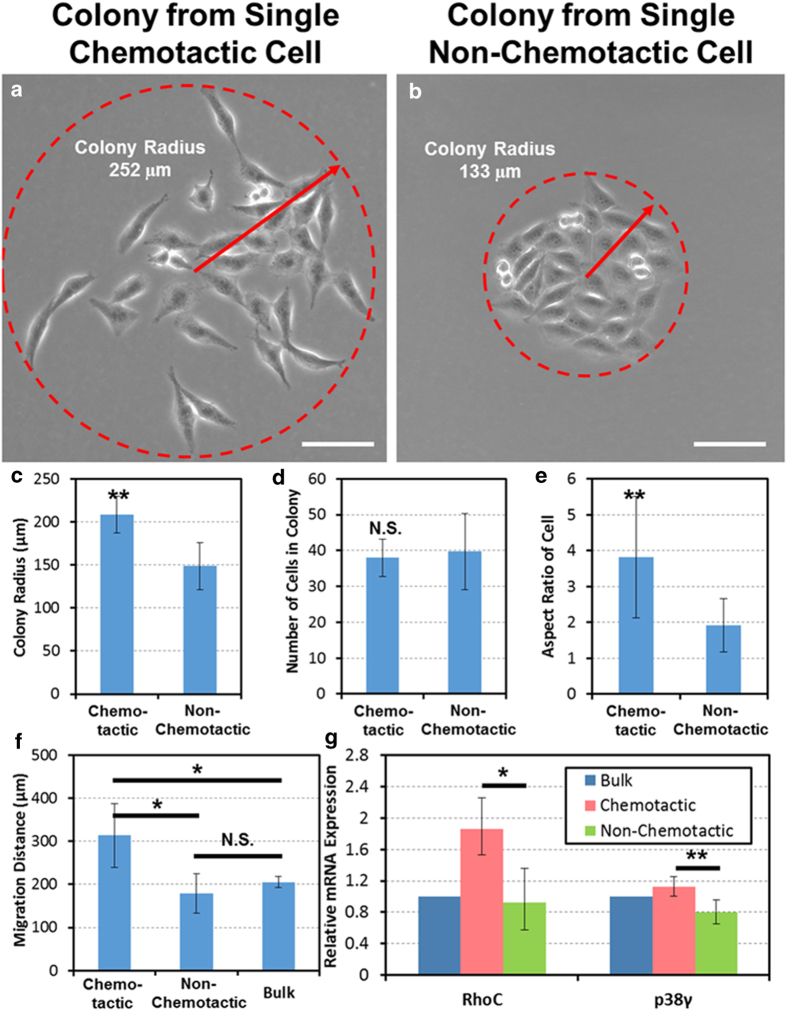
Characterization of the highly chemotactic subpopulation. (**A**) The colony formed by a single chemotactic cell after 4 days. The cells in the colony maintained an elongated (mesenchymal-like) shape and spread over a wide area. (scale bar: 100 μm) (**B**) The colony formed by a single non-chemotactic cell after 4 days. The cells were epithelial in morphology and tightly clustered. (scale bar: 100 μm) (**C**) The comparison of the colony radius between the highly chemotactic and non-chemotactic cell colonies. The colonies formed by chemotactic cells have a significantly larger radius (N = 8 colonies), ** refers to P < 0.01. (**D**) The comparison of the number of cells per colony between the highly chemotactic and non-chemotactic cell colonies. No significant difference was observed. (N = 8 colonies) (**E**) The descendant cells from chemotactic cells exhibit a significantly higher aspect ratio 4 days after retrieval, indicating persistence of the mesenchymal-like morphology (N = 8 colonies), ** refers to P < 0.01. (**F**) The migration distance of repeated single-cell migration assays. Descendants of highly chemotactic cells persisted to be more migratory than the descendants of non-chemotactic cells and the unsorted bulk MDA-MB-231 cells (N = 5 devices), * refers to P < 0.05. (**G**) The chemotactic cells exhibited a higher expression of mRNA as analyzed by qRT-PCR of the migration and metastasis-associated genes RhoC and p38γ as compared to non-chemotactic cells; * refers to P < 0.05, and ** refers to P < 0.01, respectively.

In order to evaluate the two populations further, we allowed the harvested cells to grow in tissue culture for 4 days after retrieval. The collected cells grew into distinct colonies each containing about 30–40 cells as shown in Fig. 4 (A, B, D). We found that the harvested highly chemotactic cells maintained their mesenchymal morphology, even after forming a colony over 4 days, while in comparison, the non-chemotactic cells remained tightly clustered and epithelial in appearance (Fig. 4 (A,B)). In the chemotactic colonies, all daughter cells were also spread over a larger area than those in colonies formed by non-chemotactic cells. There was a significant difference in the colony radius between the two groups (Fig. 4 (C)), with no observed difference in the proliferation rate (Fig. 4 (D)). The cells of a chemotactic colony also had significantly greater aspect ratios than cells in a non-chemotactic colony, indicating maintenance of the mesenchymal and epithelial morphologies, respectively (Fig. 4 (E)).

To examine whether the difference in chemotaxis was maintained after cell retrieval and culture, single-cell migration assays were performed on the daughter cells from the chemotactic and non-chemotactic clusters. Despite only having a limited (<1,000) number of descendant cells from the even smaller number of harvested chemotactic and non-chemotactic cells, our single-cell migration chip could efficiently handle such limited quantities due to a high capture efficiency. The progeny of the highly chemotactic cells remained significantly more migratory than those of either the harvested non-chemotactic cells or the non-migration-sorted bulk population (Fig. 4 (F)), while no significant difference was observed between the descendants of the non-chemotactic cells and unsorted MDA-MB-231 cells. These results demonstrate that the distinct characteristics of sorted cells are maintained after the harvesting and limited propagation process, allowing further studies on the differences between these highly chemotactic and non-chemotactic cells to be reliably interpreted.

Taking advantage of this fact and to further ascertain what molecular differences between these two populations within the same cell line might account for their different migration behavior, we harvested the chemotactic and non-chemotactic populations from a separate set of migration devices and cultured them for 4 days as before. We then isolated RNA from the chemotactic and non-chemotactic cells as well as from bulk MDA-MB-231 cells plated in the similar numbers to the quantity harvested from the migration devices. We performed RT-qPCR on samples from 3 separate chemotaxis-sorting experiments and found that the chemotactic cells expressed significantly higher amounts of RhoC GTPase and p38γ mRNA as compared to the non-chemotactic cell population that remained on the cell-loading side of the device (Fig. 4 (G)). Both of these proteins have been shown in previous studies to be important mediators of cancer cell motility and higher expression correlated with advanced cancer stage and worse prognosis^38^,^51^,^52^,^53^,^54^.

### Customized migration channel for mimicking lymphatic capillary geometry

Capillary intravasation is a critical step in the metastasis of cancer to the lymph nodes^3^, yet limited devices at present allow for the ideal *in vitro* study of this process^15^. To address this gap, we designed a device with migration channels with a series of choke points in order to mimic the geometric constraints of lymphatic capillaries within our migration chip^26^.

**Figure 5:**
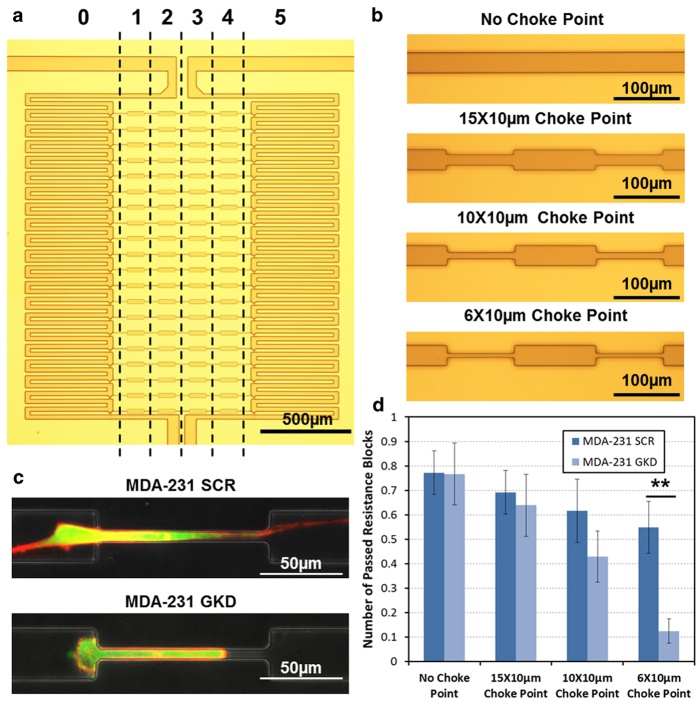
Customized migration channels for mimicking lymphatic capillary geometry. (**A**) Photomicrograph of the fabricated device. (**B**) Size variation of migration channels. The length of choke point is 100 μm, and the width of choke point varies from 6 μm (narrowest) to 30 μm (no choke point). (**C**) Qualitative observation of migration behavior of MDA-MB-231 cells in the 6 μm × 10 μm choke point. The scrambled control (SCR) cells can form a stable and long stress fiber to migrate through the choke point, while the p38γ knockdown (GKD) cells can only squeeze into the choke point. F-actin is labeled by RFP and GFP is expressed by the targeted or scrambled shRNA plasmid. (**D**) Single-cell migration assay on different channel geometries. The motilities of both cells are similar in the straight channels, but the SCR MDA-MB-231 cells are far more motile in narrower choke point channels. Data points represent means ± standard deviations (N = 8 devices), ** refers to P < 0.01.

In previous work, we studied mitogen activator of protein kinase (MAPK) family member, p38γ, which has a known role as a motility regulator in aggressive breast cancer cells^38^. In that study, MDA-MB-231 breast cancer cells transfected with shRNA targeting the p38γ isoform, p38γ knockdown (GKD) cells, had a rounded morphology and decreased or eliminated mesenchymal migration. These cells were more epithelial and had dysfunctional actin cytoskeleton cycling leading to ineffective random walk in 2-dimensional migration when compared to cells transfected with a scrambled shRNA control plasmid (SCR)^38^. Importantly, these results had been modeled previously by us *in silico* and, for the p38γ knock-down cells, the endpoint of decreased lymph metastases have been observed *in vivo*. Now, we wished to test whether our newly developed device in our present work would reliably approximate lymphatic invasion as a rapid and convenient *in vitro* mimic. Thus, to characterize the migration capability of MDA-MB-231 breast cancer cells in a 3D geometric model of lymphatic capillaries, we altered our single-cell migration chip to contain multiple migration resistance choke points (Fig. 5 (A,B)) and successfully demonstrated the capability of tracing single cells in this lymphatic capillary invasion assay (Fig. 5 (C,D)). The size variation of the migration channels and choke points are illustrated in Fig. 5 (B) and represent the continuum of mechanical stresses a cancer cell would encounter *in vivo*, with the narrowest choke point corresponding to the average diameter of lymphatic vessels draining to axillary lymph nodes^26^,^27^,^28^.

The qualitative effects of p38γ knockdown on migration through choke points can be seen in Fig. 5 (C). Figure 5 (C) shows the representative morphologies of the scrambled vector (SCR) cells and p38γ knockdown (GKD) cells within the narrowest choke point channels (6 μm × 10 μm). F-actin fibers are labeled with red fluorescence protein (RFP) and stably transfected cells are labeled with green fluoresecence protein (GFP) through their respective expressing plasmids. SCR cells are able to form long pseudopodia that can reach past the choke point in a mesenchymal-like manner to form an attachment beyond the narrowing; thus, these cells are successful in migrating through the lymphatic-capillary mimics by contraction of their stress fibers in the typical “rubber band-like” fashion (Fig. 5 (C)). In contrast, the GKD cells ineffectively cycle their cytoskeleton in a basket-weave configuration, as demonstrated previously^38^, and thus are only able to squeeze into the narrow channel but can travel no further, with the actin evenly distributed around the periphery of the cell (Fig. 5 (C)).

To quantify this observation further, we measured the cell migration distance (as a function of “passed choke points” or relative distance in the channels) for multiple choke point geometries, as plotted in Fig. 5 (D). We observed that SCR and GKD cells have equivalent chemotactic potential in response to a serum gradient when the migration channel is wide (30 μm × 10 μm) and without choke points, but the number of traversed choke points of GKD cells significantly diminishes when the migration is obstructed by the narrowest choke points (6 μm × 10 μm). To verify the decreased migration efficiency of p38γ knockdown cells, the migration velocity of MDA-MB-231 cells in the narrowest choke point channels was measured and shown in Fig. S5. While the variation is large, the migration velocity of SCR cells is almost double that of GKD cells. This result supports the hypothesis that the motility of GKD cells is decreased due to unproductive actin cytoskeletal cycling as previously reported^38^, but also allows, for the first time, direct visualization of what might be happening *in vivo* at the critical intravasation step for lymph node metastases that caused the GKD cells to have decreased contralateral lymph node metastases in mice^38^.

## Discussion

Many microfluidic devices for cell migration have been reported in recent years^14^,^15^,^16^,^25^,^32^,^33^,^34^,^35^,^36^,^37^,^40^,^41^. Although multiple approaches have been designed to exploit the advantages of microfluidics (small volumes and precise micro-environment control), most assays still measure an average behavior over large numbers of cells with an underlying implicit assumption that all cells are essentially identical. However, as cellular heterogeneity is increasingly recognized as a key aspect of the evolution of cancers and of the genesis of inherent resistance to treatment and recurrence^29^, there is a need to leverage microfluidics for the study of tumor heterogeneity. In this work, we have developed, characterized, and tested a tool that has the capability to select highly chemotactic cells for study and to enable their recovery for further characterization of this subpopulation’s differences from its non-chemotactic counterpart population. Given that only certain cells within a tumor are the key metastases-initiating cells^1^,^2^, we anticipate that this tool has the potential to greatly advance our detailed molecular studies of the multiple cellular subpopulations comprising a primary or a metastatic tumor. Understanding specific differences that lead some cancer cells to leave the primary tumor and seed metastases is of great benefit to develop and test anti-metastatic strategies. Here, we demonstrated single-cell migration and investigation of the individual chemotactic profile of each cell rather than their average aggregate behavior. Moreover, following the assays, cell populations of different chemotactic potential extremes were selectively retrieved for further downstream analysis to better query the inherent differences in these subpopulations.

The presented migration device reliably positions exactly one cell next to the migration channel, granting the advantages of using a small number of cells and allowing for easy tracing of single-cell migration behavior. We incorporated a hydrodynamic scheme within the migration channel that, through optimization, achieved near an 85% capture rate in 5 different mammalian cell lines. In order to achieve this high single cell capture efficiency, precise control of the hydrodynamic resistances was necessitated not just for the individual channel subsections (serpentine channel vs. central channel) but also across the whole migration device. When more than 20 migration channels (and thus 20+ serpentine channels and 20+ central channels) were incorporated, the gravity-driven flow rate with the volumes used for cell seeding was too slow and cell clogging occurred. However, even with just 20 migration channels, the assays can be performed on the limited inputs of cells as demonstrated by our post-assay recovery and re-assessment of migration properties of less than 1,000 cells. In addition, a chemoattractant gradient can be reliably generated in the narrow migration channels with a limited effect on the concentration profile by a migrating cell.

Using the described platform, we have successfully demonstrated three single-cell migration assays: tracing SKOV3 cell chemotaxis induced by HGF, determining molecular differences between the highly chemotactic and non-chemotactic populations of MDA-MB-231 breast cancer cells, and studying a metastasis-related gene (p38γ) by evaluating its effect on cancer cell migration through channels mimicking the geometric constraints of lymphatic capillary intravasation. Our prior work revealed the differences between GKD and SCR cells in their actin cytoskeleton oscillations and random-walk migration via computer modeling and demonstrated reduced lymphatic metastases *in vivo* in mice^38^. With our newly developed single-cell migration chip, we are able to directly visualize how the GKD cells are mechanically less capable of lymphatic intravasation. This experiment demonstrated the potential of the proposed single-cell platform for studying models of cell migration *in vitro* in devices that can geometrically mimic critical steps in the metastatic cascade and the ability to discern cellular motility differences as a result of specifically induced or native signaling characteristics. Furthermore, evaluation of the motility and indirect metastatic potential of certain cell fractions *in vitro* has the potential to enable targeting specific cell subpopulations *in vivo* so as to eliminate them preferentially before they may have a chance to metastasize.

Our platform also provides a method for chemotactic-based selection. Highly chemotactic and non-chemotactic cells were selectively retrieved after the migration assay for further propagation and analysis. While a previously reported device also allows for the chemotactic selection of cells^16^, this device suffers from the necessity of loading thousands of cells precluding its potential use with small tumor biopsy samples. Utilizing our device and with loading only hundreds of cells, we demonstrate that the distinct characteristics of migration-sorted cells are maintained after harvesting and limited expansion in tissue culture. This allows for reliable interpretation of further downstream studies to distinguish the differences between the highly chemotactic and non-chemotactic cell populations within a given “homogenous” sample. Thus, the present platform provides the capability to correlate the migration phenotype of the highly chemotactic cells with a molecular signature of gene expression within this subpopulation. Although a recent study demonstrated poor correlation between the speed of mother and daughter cells in the immediate 6 hours after cell division, this was for cells from the entire spectrum of migration speeds^14^. In our work, we select for the highly chemotactic subpopulation and show that for these cells this migration and mesenchymal morphology phenotype is heritable, at least over limited generations. Furthermore, this same population of cells also expresses greater amounts of mRNA of two known migration and metastasis-associated genes, RhoC and p38γ, than the non-chemotactic subpopulation does^38^,^51^,^52^,^53^,^54^. Therefore, our present device has the capability of selecting cancer cells based upon their chemotactic phenotype and then enabling the harvest and assessment of what molecular underpinnings might be responsible for the difference in chemotaxis. We believe our study sets the stage for the investigation of motility heterogeneity and metastatic potential within cancers on a broader scale and can yield new insights as to the mechanical and molecular basis of why certain tumor cells in a patient are able to metastasize.

## Methods

### Device Fabrication and Assembly

The migration devices were formed from a single layer of PDMS (polydimethlysiloxane), which was fabricated on a silicon substrate by standard soft lithography, and a glass slide. Three masks were used to fabricate the multiple heights for the serpentine channel region (40 μm height), the capture gap (20 μm height), and the migration channel (10 μm height). Channel widths were 40 μm unless otherwise stated (choke points and central path). The PDMS layer was bonded to the glass slide after activated by oxygen plasma treatment (80 Watts, 60 seconds) to form a complete fluidic channel. Before cell loading, collagen (Collagen Type 1, 354236, BD Biosciences) solution (1.45 mL Collagen, 0.1 mL acetic acid in 50 mL DI Water) was flowed through the device for one hour to coat collagen on the substrate to enhance cell adhesion. Devices were then rinsed with PBS (Gibco 10082) for one hour to remove the residual collagen solution before use.

### Cell Culture

SKOV3 (ovarian cancer) and A2780DK (ovarian cancer) cells were obtained from Dr. Ronald Buckanovich’s lab (University of Michigan, MI, USA) and cultured in RPMI (Gibco 11875) with 10% FBS (Gibco 10082) and 1% penicillin/streptomycin (Gibco 15140). PC3 (prostate cancer) cells were obtained from Dr. Ken Pienta’s lab (University of Michigan, now at Johns Hopkins University) and cultured in DMEM (Gibco 11965) with 10% FBS and 1% penicillin/streptomycin. MDA-MB-231 (breast cancer) cells were cultured in RPMI with 10% FBS and 1% penicillin/streptomycin. Cells were cultured at 5% CO_2_. The p38γ knockdown MDA-MB-231 (GKD) cells were stably transfected in the Merajver lab with short hairpin RNA (shRNA) targeting p38γ and the scrambled vector (SCR) cells were transfected with a scrambled hairpin RNA as previously reported^45^.

### Single-cell Migration Assay

Cells were harvested from culture plates with 0.05% Trypsin/EDTA (Gibco 25200) and centrifuged at 1000 rpm for 5 min. To improve the imaging quality, cells were stained by green fluorescent (Invitrogen, Cell tracker Green C2925) dye. Then, the cells were re-suspended in culture media to a concentration of 1 × 10^5^ cells/ml. 100 μL of this cell solution was pipetted into the left inlet, and 80 μL of media only was pipetted into the right inlet. After 10 minutes, the cell solution in the left inlet was replaced with 100 μL serum-free culture media, and 100 μL serum-free media with the indicated chemoattractant was applied to the other inlet to induce chemotactic migration. Then, the entire chip was put into a cell culture incubator. Migration distance was measured based on the final cell position after 24 hours of incubation without media replenishment. Only the migration channels having single cells were counted. The velocity of cells was measured by imaging cell positions every 30 minutes. Results presented represent means ± standard deviations. A two-tailed student t-test (unpaired) was used to measure significance.

### Selective Subpopulation Retrieval

We selectively harvested the chemotactic and non-chemotactic cells after a 24 hour migration assay. The cells that remained in the left side (loading side) were considered non-chemotactic cells, while the cells that had migrated the entire 1 mm migration channel to the right side (chemoattractant side) within 24 hours were considered highly chemotactic cells (Fig. 3(A)). To avoid possible contamination, both the inlets and outlet were washed carefully with trypsin before cell harvesting. 100 μL of PBS was pipetted into the outlet and left for 5 minutes to wash away any residual serum or debris in the channel. Trypsin was then pipetted into each outlet and flowed from the outlet to the inlet for 5 minutes. In this manner, the highly motile cells were trypsinized and directed to the right inlet, while the non-motile cells were directed to the left inlet. A slight negative pressure (~1,000 Pa) was also applied to each collecting inlet to prevent cell capture by the central paths since with this increased flow rate the cells overcame the slight difference in diameter and deformed and flowed through the central path. The detached cells were pipetted into a 60 mm petri dish or 96-well plate for recovery and propagation. As cells plated at an ultralow density (~tens of cells) have poor viability, we cultured these cells in MDA-MB-231 conditioned media. Conditioned media was obtained by culturing 3 mL of RPMI supplemented with 10% FBS on a 80% confluent layer of MDA-MB-231 in a 60 mm dish for one day prior to harvesting. Three consecutive 10 minute, 2,000 rpm centrifuging processes removing and re-centrifuging the media supernatant were performed to remove possible cellular contamination from the conditioned media once it was removed from the cell conditioning plate. The triple-centrifuged conditioned media was further plated alone and cultured in an incubator as a control to verify that no residual MDA-MB-231 cells were introduced into our chemotactic and non-chemotactic cultures.

### RNA Isolation and RT-qPCR

After selective subpopulation retrieval, the harvested chemotactic and non-chemotactic cells were plated into 96-well plates for 4 days of culture. MDA-MB-231 cells that had not been migration-sorted were plated in equal densities to the chemotactic and non-chemotactic populations and used as bulk control. Biological replicates were harvested from 3 separate migration sortings each comprising at least 10 devices. RNA was extracted using the Single Cell RNA Isolation Kit (Norgen Biotek Corp, Cat. 51800) according to the manufacturer’s standard protocol. RNA was eluted in 9 μL. The cDNA was prepared using the Reverse Transcription System (Promega, Cat. A3500) according to the standard protocol using Oligo(dT) primers and the entirety of the harvested RNA (~7 μL) was used in each 20 μL cDNA reaction. The reaction was incubated at 42 °C for 45 minutes then inactivated at 95 °C for 5 minutes before storing at -20 °C until use. Template dilutions of the 20 μL cDNA reactions were made by mixing 10 μL of cDNA with 1 μL of RNase/DNase free water. Qiagen QuantiTect SYBR Green PCR Kit (Cat. 204143) was used for the qRT-PCR reaction according to the standard manufacturer’s hot-start protocol. Primers were purchased from Integrated DNA Technologies: RPL22 (Hs.PT.51.607028), RPL30 (Hs.PT.51.3119226), RhoC (Hs.PT.56a.39081600), and p38γ (Hs.PT.58.45504579). Primers were diluted into 500 μL of TE buffer (20x). Each 20 μL reaction well contained 10 μL of mastermix, 8 μL of water, 1 μL of the 1:2 diluted cDNA, and 1 μL of 20x primer. Triplicate technical replicates were performed on each gene for each of the triplicate biological replicates. The reaction was run on a StepOnePlus Real-Time PCR System (Applied Biosystems) with melt curve analysis for specificity of products. Results were analyzed with REST 2009 software using both RPL22 and RPL30 for normalization and with 5,000 iterations^55^.

### Statistical Analysis

Two-tailed, unpaired student’s t-tests were used for all comparisons with a significance level of 0.05 considered statistically significant. For RT-qPCR results, REST 2009 analysis software was used to assess significance using both RPL22 and RPL30 for normalization and with 5,000 iterations^55^. Results are presented as mean ± SD.

## Author Contributions

Y.C. and E.Y. developed the initial concepts with input from S.G.A. and S.D.M. for the lymphatic capillary device; Y.C., S.G.A., S.D.M., and E.Y. designed the microfluidic devices; Y.C. fabricated the devices; Y.C. and S.G.A. performed the experiments; R.B. contributed key reagents and materials; Y.C. and S.G.A. analyzed data; Y.C., S.G.A., S.D.M, and E.Y. interpreted results; Y.C., S.G.A., P.N.I., S.D.M., and E.Y. co-wrote and revised the manuscript. E.Y. and S.D.M. supervised the study. All authors discussed the results and commented on the manuscript.

## Additional Information

**How to cite this article**: Chen, Y.-C. *et al*. Single-cell Migration Chip for Chemotaxis-based Microfluidic Selection of Heterogeneous Cell Populations. *Sci. Rep.***5**, 9980; doi: 10.1038/srep09980 (2015).

## Supplementary Material

Supplementary Information

Supplementary Information

## Figures and Tables

**Table 1:** Capture rates of five cell lines. (N = 4 devices).

	**Cell Type**	**Capture Rate**
Skov3	Ovarian cancer	93.8 ± 6.4%
A2780DK	Ovarian cancer	88.6 ± 10.2%
C2C12	Mouse muscle myoblast	92.2 ± 4.5%
MDA-MB-231	Breast cancer	91.5 ± 7.2%
PC3	Prostate cancer	85.1 ± 9.7%
