# Supplementary material for: Single-cell Migration Chip for Chemotaxis-based Microfluidic Selection of Heterogeneous Cell Populations
Source: Sci Rep. 2015 May 18;5:9980. doi: 10.1038/srep09980 (PMC4435023; doi:10.1038/srep09980)
Supplement: Supplementary Information [file srep09980-s1.pdf]

Title

Single-cell Migration Chip for Chemotactic-based Microfluidic Selection of Heterogeneous Cell Populations

Authors

Yu-Chih Chen[#a], Steven G. Allen[#b,c], Patrick N. Ingram[d], Ronald Buckanovich[e], Sofia D. Merajver*[b,f] and Euisik Yoon*[a,c]

[a]Department of Electrical Engineering and Computer Science, University of Michigan, Ann Arbor,MI

[b]Program in Cellular and Molecular Biology, University of Michigan Medical School, Ann Arbor, MI

[c]Medical Scientist Training Program, University of Michigan Medical School, Ann Arbor, MI

[d]Department of Biomedical Engineering, University of Michigan, Ann Arbor, MI

[e]University of Michigan Comprehensive Cancer Center, Ann Arbor, MI

[f]Department of Internal Medicine and Comprehensive Cancer Center, University of Michigan, Ann Arbor, MI

[#]Equal contribution as first authors
*Equal contribution as senior authors, corresponding authors

Euisik Yoon
1301 Beal Avenue, Ann Arbor, MI 48109-2122, USA
Tel: 734-615-4469; E-mail: esyoon@umich.edu.

Sofia D. Merajver
1500 E. Medical Center Drive, Ann Arbor, MI 48109, USA
Tel: 734-936-6884; E-mail: smerajve@umich.edu

Supporting Information

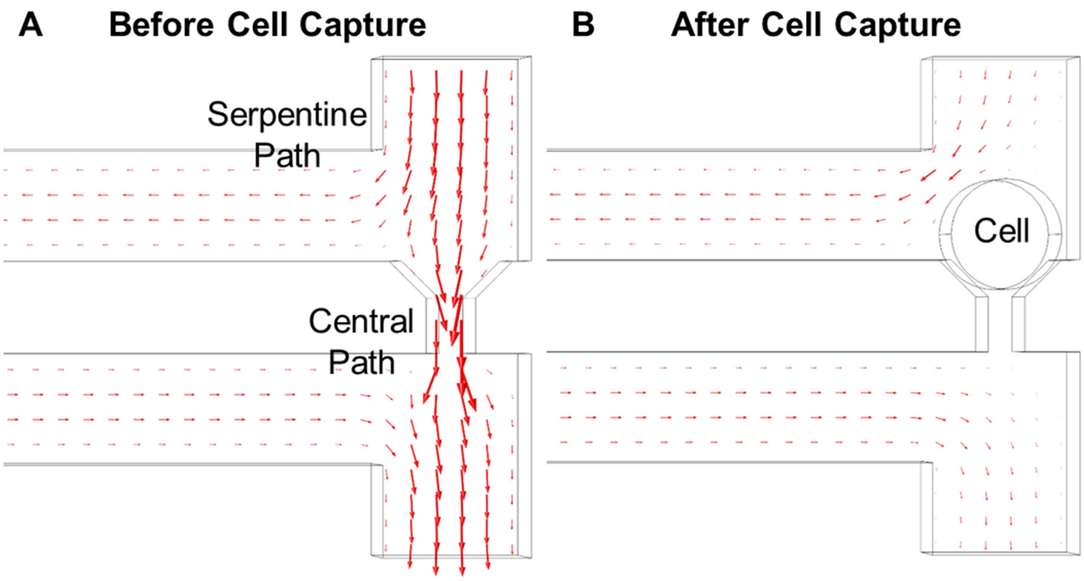

Fig. S1. Simulations of flow velocity before and after capturing one cell by COMSOL 4.3: (A) Before cell capture, simulation of flow velocity shows that the higher flow rate through the central path, so the cells will more likely follow the central path. (B) After capturing one cell, the captured cell plugs the gap and blocks the flow through the central path. Thus, the rest of the cells will travel through the serpentine path and will be subsequently captured in the downstream capture sites.

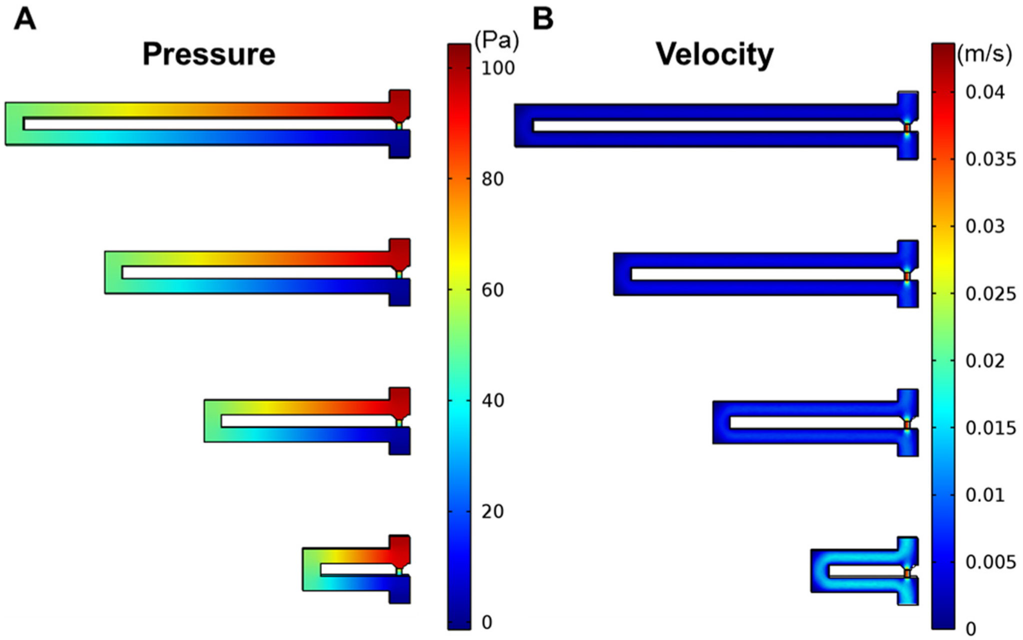

Fig. S2. Simulations of flow velocity and pressure on different serpentine lengths ranging from 200 µm to 800 µm by COMSOL 4.3: (A) Simulations of pressure distribution illustrates that the quick transition in the capture site leads to a high capture probability of single cells at the site. (B) Simulations of flow velocity indicates that when the serpentine structure is short, the flow rate through serpentine path becomes higher, which means that the cell is less likely to be driven to the capture gap.

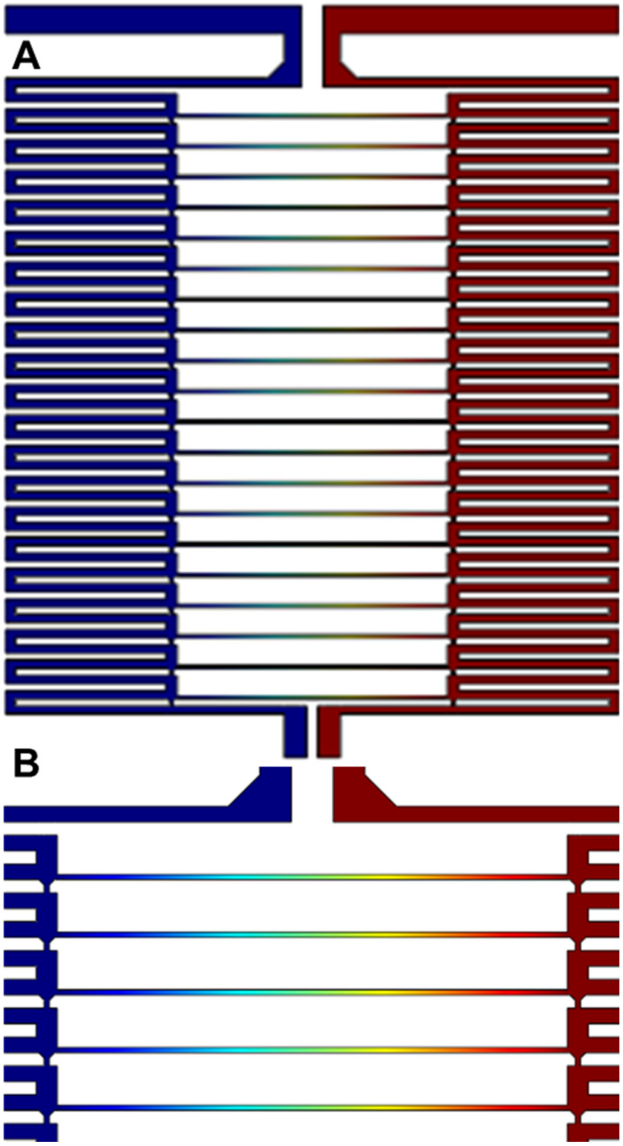

Fig. S3. Simulations of chemical concentration gradient generated in the device by COMSOL 3.5. (A) The simulation of the whole chip demonstrates that the chemical concentration is uniform from the upstream to the downstream channels since the diffusion is relatively slow. (B) Enlarged view of the first few channels. The simulated concentration profile shows the linear chemical gradient is formed in the migration channel. Concentrations are shown in color scale with red being 1 M chemokine and blue being 0 M chemokine.

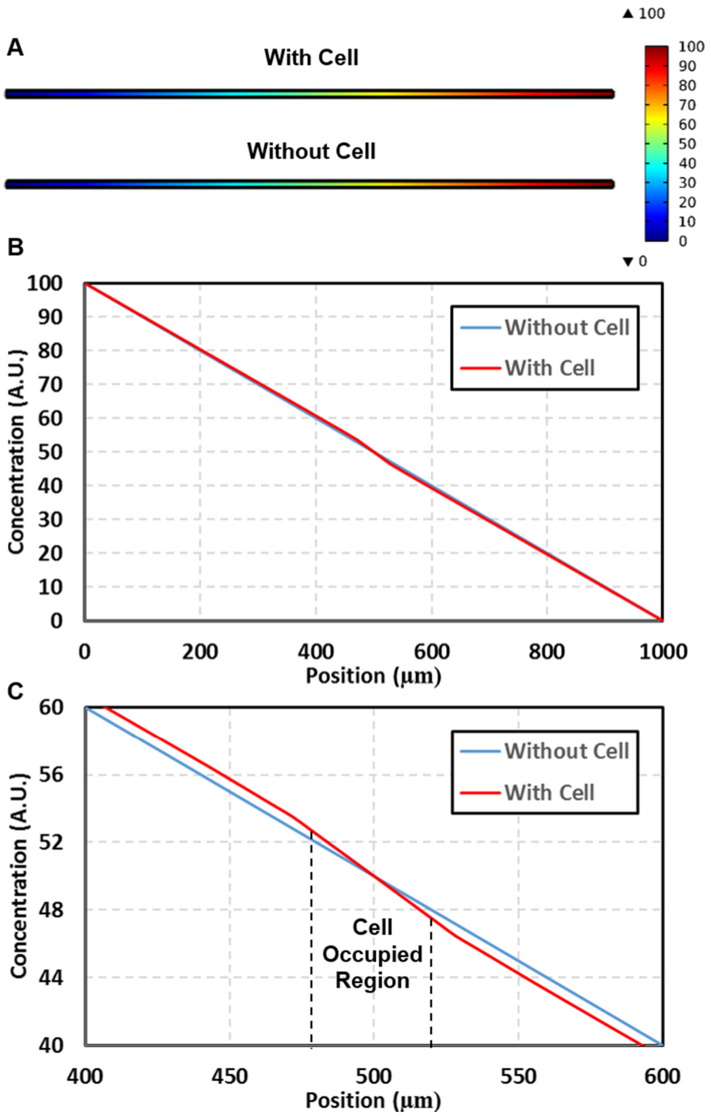

Fig. S4. Simulations of chemical concentration profile with and without cell migrating in the migration channel. (A) The simulation of the chemical concentration profile with and without cell migrating in the migration channel. The cell was emulated by adding a pseudo-cell (10 µm width by 10 µm height and 40 µm length) on the bottom of the channel to block diffusion. The cell was placed at the center (500 µm from the left) (B) The concentration profile in the channel. (C) Enlarged concentration profile in the channel from the 400 µm to 600 µm position. Since the channel cross-section (10 µm by 40 µm) is much larger than cell, the concentration is altered by less than 2%.

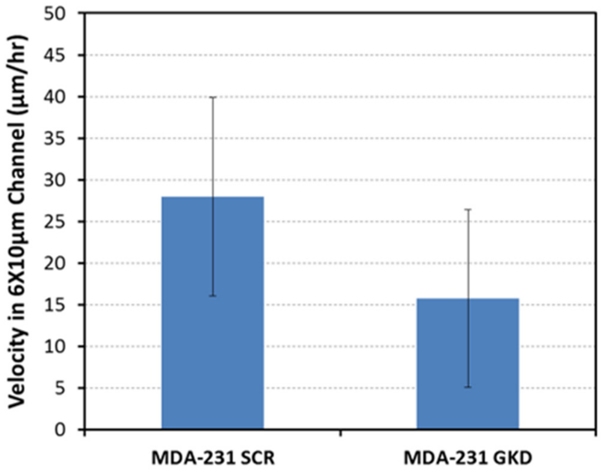

Fig. S5. The migration velocity of MDA-MB-231 cells in the 6 µm x 10 µm choke points. The scrambled control (SCR) cells can migrate more efficiently than the p38γ knockdown (GKD) cells through the choke point.

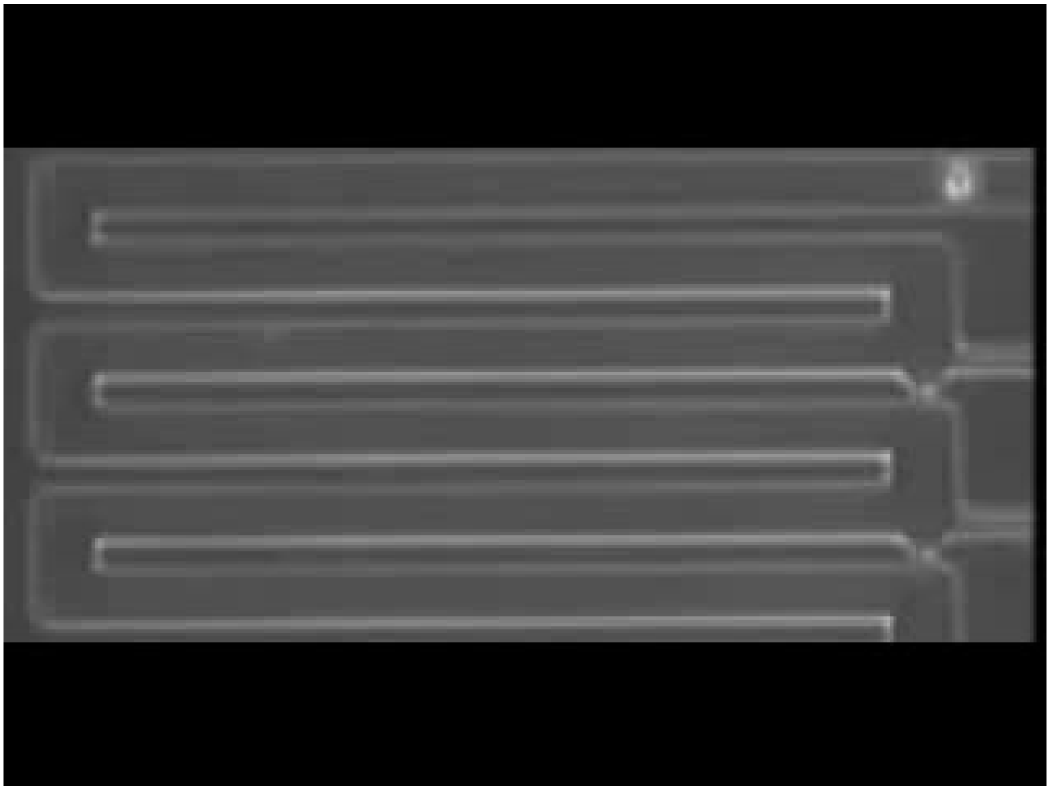

Mov. S1. The single-cell capture process in the presented migration chip.
